# Leveraging Mobile Phone Sensors, Machine Learning, and Explainable Artificial Intelligence to Predict Imminent Same-Day Binge-drinking Events to Support Just-in-time Adaptive Interventions: Algorithm Development and Validation Study

**DOI:** 10.2196/39862

**Published:** 2023-05-04

**Authors:** Sang Won Bae, Brian Suffoletto, Tongze Zhang, Tammy Chung, Melik Ozolcer, Mohammad Rahul Islam, Anind K Dey

**Affiliations:** 1 Human-Computer Interaction and Human-Centered AI Systems Lab AI for Healthcare Lab, School of Systems and Enterprises Stevens Institute of Technology Hoboken, NJ United States; 2 Department of Emergency Medicine Stanford University Stanford, CA United States; 3 Institute for Health Healthcare Policy and Aging Research Rutgers University Newark, NJ United States; 4 Information School University of Washington Seattle, WA United States

**Keywords:** alcohol consumption, binge-drinking event, BDE, behavioral prediction model, machine learning, smartphone sensors, passive sensing, explainable artificial intelligence, XAI, just-in-time adaptive interventions, JITAIs, mobile phone

## Abstract

**Background:**

Digital just-in-time adaptive interventions can reduce binge-drinking events (BDEs; consuming ≥4 drinks for women and ≥5 drinks for men per occasion) in young adults but need to be optimized for timing and content. Delivering just-in-time support messages in the hours prior to BDEs could improve intervention impact.

**Objective:**

We aimed to determine the feasibility of developing a machine learning (ML) model to accurately predict *future*, that is, *same-day BDEs 1 to 6 hours prior BDEs*, using smartphone sensor data and to identify the most informative phone sensor features associated with BDEs on weekends and weekdays to determine the key features that explain prediction model performance.

**Methods:**

We collected phone sensor data from 75 young adults (aged 21 to 25 years; mean 22.4, SD 1.9 years) with risky drinking behavior who reported their drinking behavior over 14 weeks. The participants in this secondary analysis were enrolled in a clinical trial. We developed ML models testing different algorithms (eg, extreme gradient boosting [XGBoost] and decision tree) to predict same-day BDEs (vs low-risk drinking events and non-drinking periods) using smartphone sensor data (eg, accelerometer and GPS). We tested various “prediction distance” time windows (more proximal: 1 hour; distant: 6 hours) from drinking onset. We also tested various analysis time windows (ie, the amount of data to be analyzed), ranging from 1 to 12 hours prior to drinking onset, because this determines the amount of data that needs to be stored on the phone to compute the model. Explainable artificial intelligence was used to explore interactions among the most informative phone sensor features contributing to the prediction of BDEs.

**Results:**

The XGBoost model performed the best in predicting imminent same-day BDEs, with 95% accuracy on weekends and 94.3% accuracy on weekdays (*F*_1_-score=0.95 and 0.94, respectively). This XGBoost model needed 12 and 9 hours of phone sensor data at 3- and 6-hour prediction distance from the onset of drinking on weekends and weekdays, respectively, prior to predicting same-day BDEs. The most informative phone sensor features for BDE prediction were time (eg, time of day) and GPS-derived features, such as the radius of gyration (an indicator of travel). Interactions among key features (eg, time of day and GPS-derived features) contributed to the prediction of same-day BDEs.

**Conclusions:**

We demonstrated the feasibility and potential use of smartphone sensor data and ML for accurately predicting imminent (same-day) BDEs in young adults. The prediction model provides “windows of opportunity,” and with the adoption of explainable artificial intelligence, we identified “key contributing features” to trigger just-in-time adaptive intervention prior to the onset of BDEs, which has the potential to reduce the likelihood of BDEs in young adults.

**Trial Registration:**

ClinicalTrials.gov NCT02918565; https://clinicaltrials.gov/ct2/show/NCT02918565

## Introduction

### Background

Binge-drinking events (BDEs), defined as consuming ≥4 drinks on a single occasion for women or ≥5 drinks on a single occasion for men (National Institute on Alcohol Abuse and Alcoholism), represent a common, risky drinking pattern, primarily seen among young adults (aged 18 to 25 years; Centers for Disease Control and Prevention). BDEs are associated with multiple adverse alcohol-related harms, such as traffic fatalities and violent behavior [1,2]. Digital behavioral interventions demonstrate some promise in reducing hazardous alcohol consumption among young adults [3-6]. Digital behavioral interventions using various communication modalities (eg, app, text messaging, and interactive voice response) can deliver content and collect responses over time, potentially adapting the timing and content of the intervention to specific contexts and needs (eg, motivational enhancement after self-regulation failure) [7]. Systematic reviews of digital behavioral interventions generally find modest effects on reducing unhealthy alcohol use [3-6], suggesting the need to boost the effectiveness of technological interventions. One of the ways to potentially increase the effects of digital intervention is to provide just-in-time (JIT) support when it will have an optimal impact [8] to modify drinking intentions and behavior.

### Smartphone-Based Sensors to Detect BDEs

Timely intervention delivery depends on the ability to accurately predict the imminent (ie, 1 to 6 hours prior) occurrence of a BDE. To date, most research has focused on the simpler problem of “detecting” an episode of alcohol use that has already started. For example, sensors (eg, transdermal alcohol sensor) worn on the wrist or ankle that use biological sampling can “detect” a BDE [9,10] and provide “ground truth” for identifying BDEs but cannot “predict” a BDE that has not yet started. Regarding the topic of detection, a study using cognitive tasks performed on a smartphone [11] demonstrated that it is possible to estimate (ie, similar to detection) one’s blood alcohol level from their ability to perform cognitive tasks on a smartphone (eg, text entry analysis, swiping on screen, and balancing). Similarly, mobile systems such as the Drunk User Interfaces (DUI) app [11] measure how alcohol affects motor coordination [12] but have limited ability to *predict* future episodes of alcohol use.

Smartphone sensors provide potentially powerful tools for collecting continuous data that can be used to infer personal behaviors or activities associated with alcohol use with low burden and at low cost. The smartphone’s accelerometer has been used to detect or measure alcohol-related intoxication through gait analysis [13-15]. In addition, mobile crowdsensing using smartphone sensor data (eg, GPS and Bluetooth) has been used to “detect” or classify drinking episodes in youth on weekend nights [16]. Similarly, our group’s work using smartphone sensors to detect alcohol use in young adults found that the most informative features involved time (eg, day of week), movement (change in activities), and communication (call duration) [17,18]. These “detection” studies focused on alcohol use that already started. However, to prevent the onset of a BDE, a model that predicts imminent (eg, same-day) BDE is needed so that JIT support can be delivered before the onset of drinking.

To date, most prediction models of imminent drinking and BDEs are based on self-report. For example, the A-CHESS smartphone app is a JIT adaptive intervention (JITAI), which used weekly check-ins to track alcohol-dependent patients’ recovery progress (ie, a risk score based on 5 items, such as urge, depression, and sleep problems), with a Bayesian network prediction model of lapse risk (any alcohol or illicit drug use) in the coming week that had 75% sensitivity and 88% specificity [19]. Other studies using ecological momentary assessments (EMAs) to predict substance use found, for example, that negative affect, stress, and craving or urge to drink were important predictors of substance use in patients who completed substance use treatment [20], adults experiencing homelessness [21], and emerging adults who reported drinking [22]. Although these mobile apps and EMA studies demonstrate the utility of self-report in predicting imminent episodes of substance use, EMAs can be burdensome to complete, especially if multiple reports per day are required over a long duration.

### Limitations of the Existing Studies on Mobile Sensor–Based Alcohol Detection

Smartphone sensors provide a low-burden method to support a continuous stream of data for a prediction model of imminent substance use. However, few studies have used smartphone sensors to “predict” substance use. Models for predicting substance use differ from the models for detecting substance use in requiring that data for prediction be collected before the onset of use and that a period (eg, 1 hour) separates the predictors (eg, phone sensor features) and predicted outcome (eg, drinking event). Predicting drinking events *before* they begin theoretically allows for a greater probability that the intervention material would be effective. A study that developed a prediction model of substance use craving using GPS developed a random forest model with 93% accuracy in predicting drug craving 90 minutes into the future, based mostly on predicting the absence of craving (owing to the low craving base rate) [23]. A prediction model using a variety of smartphone sensors (eg, time, GPS, and communication logs) has yet to be applied to the prediction of an imminent (same-day) drinking event. More importantly, there is a need for increased explainability of machine learning (ML) research in the medical domain to support JITAI development.

Although the ideal time to intervene before a drinking event to optimally influence drinking intentions is not currently known, the closer in time someone is to a planned event, the more difficult it will likely be to modify those plans [24]. Alternatively, the farther away from the onset of a drinking event certain interventions or suggestions (eg, managing the desire or urge to drink) are, the less relevant they might be.

### Needs for Explainability of Predictive Models and Benefits of Explainable Artificial Intelligence Methods

ML models that predict a clinical outcome often do so without providing information on the factors that contributed to outcome prediction, commonly known as the “black box” problem. Recent work has explored how explainable artificial intelligence (XAI) can provide greater transparency in how artificial intelligence generates model output. There are 2 core benefits of XAI methods. First, XAI provides transparency into factors contributing to the prediction of the outcome [25], which can increase trust in the model results [26]. Second, XAI enables hypothesis testing and algorithm adaptation through methods such as identifying feature importance and rule extraction [27]. XAI has been applied in the medical domain for disease prediction using visual data (eg, cancer [28] and cardiovascular disease [29]). However, the advantages of XAI, when combined with an ML prediction model, have not yet been applied to smartphone sensor data and the prediction of BDEs [30,31].

### Study Objectives and Novelty

With the success of our previous detection model, as a next step in our research, we focused on (1) developing a *prediction model* for imminent (1 to 6 hours) BDEs by *optimizing analysis windows and prediction distances* from the onset of BDEs and (2) enhancing the expandability of the algorithms by adapting XAI-generated explanations (eg, Shapley additive explanations [SHAP] and partial dependence plots [PDPs]) to explore the interactions among key features in predicting same-day BDEs to support the algorithmic *decision-making* in moving toward developing JITAIs.

To the best of our knowledge, this work is the first attempt to go beyond the detection of BDEs and predict imminent BDEs using the combination of smartphone sensor data and ML coupled with XAI for enhancing the explainability of the algorithms. The prediction model of BDEs combines ML and XAI toward the ultimate goal of supporting JITAIs to prevent BDEs [2,32-34].

The novelty of our study is two-fold: (1) building an ML prediction model of drinking events and (2) combining ML with XAI to identify the most informative phone sensor features that explain the prediction model’s performance. Notably, the best-performing prediction model of drinking events can support JITAI development by providing “windows of opportunity” and identifying “key contributing features” to facilitate a digital intervention’s algorithmic decision-making.

### Research Questions

The ability to accurately predict imminent BDEs (occurring 1 to 6 hours into the future) would support the delivery of JIT behavior change strategies [8]. The prediction model, combined with XAI, would explain which smartphone sensor features are the most informative in predicting imminent BDEs to inform JITAI development. For example, if GPS-based travel patterns predicted imminent BDEs, JIT messaging could support the reinforcement of personal drinking goal commitment and provide personalized suggestions for alternative healthy activities before alcohol consumption begins [7]. Therefore, the questions we attempt to answer are as follows:

1. Can we predict BDEs versus non-drinking and low-risk (1 to 3 or 1 to 4 drinks per occasion for women and men, respectively) drinking events using ML based on passively sensed smartphone sensor data collected before drinking onset?
2. Which smartphone sensor features contribute the most to predicting BDEs on weekend and weekdays?
3. How much data (ie, analysis time window; eg, 1 to 12 hours) collected before BDEs and what prediction distance from the onset of BDEs (eg, 1 to 6 hours) can be used to *optimize the prediction of same-day BDEs* on weekend and weekdays?
4. We explored the use of XAI visualization methods to identify factors contributing to prediction in the best performing model. XAI results can be used to inform algorithmic decision-making and support JITAI development.

## Methods

### Study Participants

We recruited 75 individuals aged 21 to 25 years with at least 1 past-month report of a BDE from emergency departments (EDs) in the greater Pittsburgh area. The ED sample was a subset of participants enrolled in a 5-arm randomized trial (ClinicalTrials.gov: NCT02918565). Methods of screening, enrollment, and clinical trial procedures are detailed in a previous study [35]. The 75 participants (n=53, 71% women; n=40, 53% White; n=23, 31% Black; n=12, 16% other race; n=8, 11% Hispanic ethnicity; n=34, 45% college enrolled) who met study inclusion criteria had an average age of 22.4 (SD 1.9) years.

### Ethics Approval

Our study underwent a full review by the institutional review board of each author's institution, and was granted an exemption for the secondary analysis by both the Stevens Institute of Technology [Stevens: 2023-018 (N)] and the University of Washington (STUDY00016480), under the grant number 1R21AA030153-01. A unique device ID was assigned to the participants. The sensor and SMS text message data were labeled with the assigned unique ID. The participants were told that they could drop out of the study at any time during the study. Researchers informed the participants that phone sensors needed to be enabled during the study. However, the participants recognized that the AWARE app and certain sensors are configurable, which means that, for example, GPS can be disabled manually by accessing the setting menu on their smartphones. To keep the sensor data confidential, the study data were deidentified, and personal identifying information (eg, name and contact information) was stored separately from other study data in a secure (eg, password protected and locked) location.

The participants provided informed consent before study participation, completed baseline questionnaires, and installed our AWARE data collection app [36] on their smartphones. They were paid US $20 for installing the data collection app and completing the baseline surveys. They were also compensated for the collection of phone sensor and phone survey data at the end of the 14 weeks (US $10 per week for each week of data collection, up to US $140).

### Mobile Sensing Software and Phone Sensor Data

**Figure 1:**
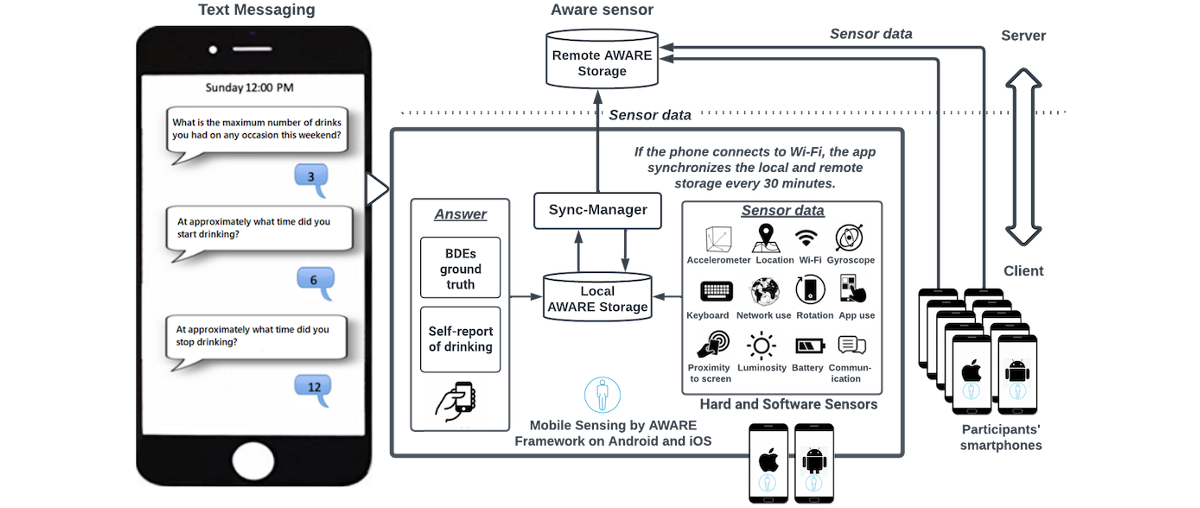
AWARE-based mobile sensing framework and text messaging—sensing app on Android (Google LLC)/iOS (Apple Inc) and drinking questions—which detail the sensor streams the app collected. BDE: binge-drinking event; iOS: iPhone operating system.

We developed our mobile data collection app based on the AWARE framework [36] to collect data from smartphone-based sensors. Our mobile app collected time-stamped data from a range of phone sensors. Figure 1 describes how our AWARE-based mobile app functioned on Android (Google LLC) or iPhone operating system (iOS ;Apple Inc) to collect data from mobile phone sensor streams. Our app generated a unique identifier on each device automatically to distinguish each participant. The app stored the sensor data on the participant’s device. Once the phone connected to Wi-Fi, the data were synchronized with our secure server using an encryption key. Our app regularly checked (at 30-minute intervals) for Wi-Fi–based internet availability.

### Self-reported Alcohol Consumption

The participants were asked to report alcohol consumption using SMS text messages twice weekly, on the 2 days of the week that they reported drinking the most in the baseline assessment [37]. To measure prior-day alcohol consumption, we used the following question: “How many drinks with alcohol did you have yesterday?” We have successfully used this single-item measure in prior studies, where the responses positively correlated with Timeline Followback measures [38]. The National Institute on Alcohol Abuse and Alcoholism’s definition of a standard drink was provided to the participants: 12 fluid ounces of regular beer, 5 fluid ounces of table wine, or 1.5 fluid ounces of distilled spirits. The participants were asked to report whether they drank alcohol within the past 24 hours, the approximate start and end time of alcohol intake, and the number of standard drinks they consumed during this time window.

To identify drinking events, we used the responses to SMS text message queries regarding the prior day’s alcohol use [39]. Table 1 shows that most BDEs occurred from Friday (n=36) and Saturday (n=52) to Sunday (n=20) and that the start time of BDEs peaked at 5 PM (Table 2).

**Table 1:** Distribution of binge-drinking events (BDEs) by day of the week (BDEs; n=122).

Number	Days of week	Frequency, n
1	Monday	2
2	Tuesday	0
3	Wednesday	5
4	Thursday	7
5	Friday	36
6	Saturday	52
7	Sunday	20

**Table 2:** Distribution of binge-drinking event start times (from midnight to 24 hours).

Number	Time of day	Frequency, n
1	00:00 to 01:00	3
2	01:00 to 02:00	0
3	02:00 to 03:00	0
4	03:00 to 04:00	0
5	04:00 to 05:00	1
6	05:00 to 06:00	0
7	06:00 to 07:00	1
8	07:00 to 08:00	4
9	08:00 to 09:00	1
10	09:00 to 10:00	3
11	10:00 to 11:00	6
12	11:00 to 12:00	3
13	12:00 to 13:00	5
14	13:00 to 14:00	2
15	14:00 to 15:00	6
16	15:00 to 16:00	9
17	16:00 to 17:00	7
18	17:00 to 18:00	15
19	18:00 to 19:00	11
20	19:00 to 20:00	12
21	20:00 to 21:00	11
22	21:00 to 22:00	8
23	22:00 to 23:00	10
24	23:00 to 24:00	4

### Drinking Prediction Model Development

To develop a model for predicting drinking events, we followed a standard ML pipeline approach: preprocessing data, preparing the data set, extracting and selecting features, and training and testing models.

#### Preprocessing Data

In total, 1168 events were reported by the 75 young adults: 729 non-drinking events (ie, days with no drinking event), 236 low-risk drinking events (ie, days on which 1 to 3 or 1 to 4 drinks per occasion were consumed by women or men, respectively), and 203 BDEs (ie, high-risk drinking days on which ≥4 or ≥5 drinks were consumed per occasion by women or men, respectively). We included the participants in our analysis if they reported at least 1 non-drinking at least 1 low-risk drinking or BDE and if there were at least 3 days with the minimum amount of phone sensor data needed for analysis. We excluded 414 events from the 75 participants (mean 5.52 events per person) because they did not provide GPS-based location data, and more than half of their key features were missing. Missing key phone sensor features might have occurred, for example, because the AWARE app allowed users to disable GPS collection. As such, we excluded days that were missing a few hours of sensor data (eg, owing to smartphone running out of battery), resulting in a final data set of 754 events from the 75 participants: BDEs (122/754, 16.2%), low-risk drinking events (143/754, 19%), and non-drinking events (489/756, 64.9%). For missing sensor values (ie, a few minutes, such as 5 or 10 minutes, of gap in data collection), we interpolated the average value between 2 adjacent sensor data points.

#### Preparing the Data Set

**Figure 2:**
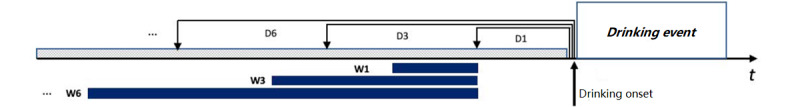
Our approach to building machine learning prediction models: “D” refers to a prediction distance from drinking onset. This prediction window varied from 1 to 6 hours. “W” refers to an analysis window, which represents the length of the time window (t) in which sensor data are being used. The analysis window varied from 1 to 12 hours.

Our strategy for drinking prediction modeling is as follows: using behavioral features from sensor data collected over a given analysis window of *w* hours (where we varied *w*), we predicted whether the participant will have a BDE within *d* hours (with varying *d* to optimize the model; Figure 2). To build this predictive model, we first created a data set containing the sensor data across the participants of the study (a data set split by “rows”; 80/20 at the level of rows), calculating features in 15-minute windows (eg, the mean of the magnitude of acceleration per minute averaged every 15 minutes). For the 15-minute window, we computed a statistical values (eg, the mean of the raw sensor) for numerical data. We encoded the time of day as a number from 0 to 23 to represent all segments of the day.

We used 15-minute segment instances as our unit of analysis because this time frame captures known social and behavioral predictors of binge drinking. The day of the week was encoded as 0 to 6. If an individual reported that they did not drink the previous day, we labeled each of the day’s 15-minute windows as a non-drinking event. When the participant reported a drinking event (BDE or low-risk drinking event), we labeled the windows before and after the drinking event as non-drinking event. For a woman, windows during a drinking event were labeled as BDE if they consumed ≥4 standard drinks, and for a man, windows during a drinking event were labeled as BDE if they consumed ≥5 standard drinks. Otherwise, the windows were coded as low-risk drinking. More than 60% (75/122; the total number of reports=122 and the number of BDE reports=75) of the BDEs reported started between 4 PM and 10 PM, as shown in Table 1, and on a Friday or Saturday, as shown in Table 2. As suggested in earlier work [40], we assumed that behavioral data aggregated during the day by smartphone-based sensors, in our case, can be applied to predict same-day drinking events.

We explored different prediction windows using 1-, 3-, and 6-hour distances prior to drinking onset to see how far in advance predictions could be made about BDEs (Figure 2). Similar to research on physical activity [41], we hypothesized that shorter window distances (eg, 1 hour) would reflect more recent behavior and would be more strongly related to same-day alcohol consumption, whereas other types of sensor data might require cumulative behavior over a longer period (eg, 6 hours of sedentary activity) to be associated with increased likelihood of alcohol use later that day. Interventions could be delivered at any of these window distances and could be tailored to be context specific at the time of delivery using a JIT framework [32]. In addition, from a technical standpoint, window size, or how much data are needed to accurately predict drinking events, impacts how much data are required to be kept on the smartphone, impacting privacy and phone storage considerations. We used window sizes of 1, 3, 6, 9, and 12 hours in our analysis. For example, if we wanted to predict whether a BDE would occur at 6 PM, we tested the predictive ability 1 versus 3 hours in advance (ie, at 5 PM vs 3 PM). Similarly, if we wanted to predict whether a BDE would occur at 3 PM, we tested the predictive ability using sensor data collected between 2 PM and 3 PM versus between 12 PM and 3 PM (ie, sensor data from 1 vs 3 hours).

We split our data into 2 nonoverlapping data sets to represent the randomly selected data set: training (80%) and testing (20%) data sets, which were split by rows. The training set was used to train ML algorithms and for model optimization. The trained model was then tested on the “unseen” data set. We used 10-fold cross-validation to identify the best model with our training data set. We report our final results on the 20% holdout test data set. To balance our data set (consisting of mostly non-drinking events), we oversampled the instances of the underrepresented classes using the synthetic minority over-sampling technique (SMOTE) [42] in our training data set, balancing BDEs and low-risk drinking events. Although SMOTE may affect the importance scores of the features we used because we filtered the features before the data were processed with SMOTE. The test data set was kept unseen and SMOTE did not affect our overall analysis.

#### Extracting Features

##### Overview

Building on our previous work in detecting BDEs with smartphone data [17,18], we extracted 70 features from smartphone sensors (Multimedia Appendix 1 [17,43]): time, location, communication (ie, calls), motion, device use, and environment. Our feature sets were calculated from the raw data based on the smartphone-based sensors and from common descriptive statistics (eg, mean and median). As there is no prior work that has identified phone sensor features associated with drinking event planning (ie, the inference of behaviors that occur before and proximal to drinking onset), we were largely exploratory in selecting features. Wherever possible, we used information from related alcohol literature to support our selection. We extracted 2 time features: day of week and time of day, as our data (Table 1) and prior studies [18,44] showed that young participants are likely to be involved in a BDE during evenings. This work also showed that more drinking events occurred between Thursday and Sunday, peaking on Saturday (Table 2).

##### Activity Level, Movement, Travel, and Location Features

The statistical features of physical activity and the magnitude (power) of body movement were extracted from the accelerometer. For location features, we chose features that have been shown to be associated with drinking behaviors [18]: total time spent at home, total number of places an individual visited, travel distance, the duration of a stay at a specific place, location entropy [45], the radius of gyration, circadian movement, location variance, the number of important places visited, and time spent at each important place. Feature extraction for circadian movement [46] was done using the Lomb-Scargle periodogram [47], which computes to what degree an individual pattern of location data follows a 24-hour circadian cycle. Location variance is the sum of the variances of latitudes and longitudes. For the number of important places visited and time spent at each important place, we used the method described by Press and Rybicki [47], which involved the clustering of the location coordinates to find important places (ie, places visited frequently). The number of locations passed in a given time (ie, travel speed) was computed from GPS data [43].

##### Communication Features

We calculated the following from call logs and contacts: the total number and duration of incoming and outgoing calls, number of SMS text messages, and total number of contacts. We hypothesized that, for example, calls and SMS text messages (logged automatically by our app) could be used to predict BDEs. We chose these features, as young adults are likely to communicate with their friends before drinking events (eg, planning to drink during the evening’s activities).

##### Device Use Features

We used screen lock and unlock events and interaction times to understand how young adults use smartphones during a day. These phone features are a potentially powerful tool to infer whether certain types of mobile phone use associated with social communication relate to near-future alcohol use (eg, phone use to arrange a meetup for a drink and finding a place using GPS and map). For device use, we used app use, screen status, and battery charging and extracted the following details from the mobile app use data: the number of unique apps used, the duration of app use, the number of times the individual switched between apps, and the number of apps running in the foreground. We extracted the following from screen status data: the number of times the screen was turned on or off and the total duration of screen use. We extracted the following details from the data on battery charging: time when the battery was fully charged, the total duration of charging battery, and battery percentages.

##### Environmental Features

Finally, we extracted the number of unique Wi-Fi hotspots as an environmental feature.

#### Building Models and Model Comparison

We built our prediction models by parsing the data for each person into 15-minute epochs. Each 15-minute epoch contained 70 sensor features and a drinking label (N: non-drinking event, D: low-risk drinking event, or BDE). We then identified all sensor features from all the 15-minute epochs in the 24 hours before the onset of a drinking event as our primary period for predictive modeling. For example, if a person reported starting drinking at 9 PM on a day they self-reported a BDE, then we would include all sensor data from the 8:45 PM–to–9 PM epoch on the previous day to the 8:45 PM–to–9 PM epoch on the current day. For days when there was no reported drinking, we randomly selected a time between 6 PM and midnight and marked this observation as the “start of the non-drinking event.” There were missing observations in the data set, so not all labeled days had ninety-six 15-minute epochs of sensor data.

As part of our modeling, we assessed the performance of the predictive models using a range of analysis window sizes (1-, 3-, 6-, 9-, and 12-hour windows) to compare the accuracies of the ML models. To test different analysis window sizes, the “prediction distance” window was held constant at “1 hour” prior to a drinking event. Note that the results indicated that a prediction window of 6 hours on weekdays and 3 hours on weekends prior to a drinking event had the best performance across analysis window sizes; therefore, the prediction windows of 6 hours on weekdays and 3 hours on weekends are reported here (refer to Multimedia Appendix 1 for results of testing analysis window sizes at other prediction distances).

We also estimated a baseline model that used only the day of the week to classify non-drinking events, low-risk drinking events, and BDEs, with the idea that a prediction model is needed to demonstrate better performance relative to baseline.

We performed hyperparameter tuning for the prediction models (separate models for weekday and weekend) for a 3-way classification task: non-drinking event (N) versus low-risk drinking event (D) versus BDE using Optuna [48]. We also trained a prediction model to predict BDE (vs N and D) for weekdays and weekends as a particularly risky form of drinking. We used popular ML classifiers and trained 5 algorithms, extreme gradient boosting (XGBoost), random forest, decision tree, support vector machine, and logistic regression, using all selected sensor features.

Hyperparameter tuning uses a search strategy to identify an optimal set of parameters that maximizes the performance of a model. To evaluate and hyperparameter tune our models, we implemented 10-fold cross-validation and then min-max scaling for feature normalization and used oversampling on the training set with SMOTE to handle class imbalance. We ran 100 iterations of Optuna to find the best hyperparameter by optimizing the *F*_1_-score of the model. As part of hyperparameter optimization, we tried different values of XGBoost. Table 3 presents the setting of the XGBoost parameters.

We chose the hyperparameter combination that achieved the best performance on the validation set for the final model that was then evaluated on the test set. To try multiple combinations of parameters, cross-validate each, and determine which values result in the best performance, we tested a variety of parameters, such as the model type, distance from the drinking events (ie, prediction window), the amount of data used (ie, analysis window size), and hyperparameters. The analysis window size was particularly important, as it defined how much data were used to fit the model. For example, an analysis window size of 3 and a prediction distance (ie, prediction window) of 6 means that the hours [17,18,49] before the event are used to predict the class of the event (N, D, and BDE).

**Table 3:** Extreme gradient boosting (XGBoost) classifier parameter settings.

Number	Function name	Parameters
1	booster	gbtree, gblinear, and dart
2	max_depth	3 to 9
3	min_child_weight	2 to 10
4	eta	1 × 10^–8^ to 1.0
5	gamma	1 × 10^−8^ to 1.0
6	grow_polic	depthwise and lossguide
7	sample_type	uniform and weighted
8	normalize_type	tree and forest
9	rate_drop	1× 10^−8^ to 1.0
10	skip_drop	1× 10^−8^ to 1.0

#### Evaluating Model Performance

To evaluate the performance of the models, we computed the following metrics to measure the overall performance: accuracy, *F*_1_-score, *κ*, precision, and recall. To measure the performance of the models across different classes, we computed precision, recall, and *F*_1_-score. In addition, we built a baseline model using only the day of the week to test how much the phone sensor features improved model performance.

First, we considered the *F*_1_-score to optimize the prediction model. The *F*_1_-score is a balanced measure of precision and recall. Second, we used the *κ* value if the *F*_1_-scores were the same. The *κ* value is a measure of the similarity between observations and predictions while correcting for agreement that happens by chance [50]. The *κ* value is also used to test performance for imbalanced classes and multiple classes.

### Feature Evaluation and Interpretation of XAI Analysis

We used the most represented XAI, SHAP [51], and PDPs [52] for the interpretation of the 2 best performing models using the XGBoost classifier for weekdays and weekends, respectively.

First, we selected SHAP [51] because it is the most common XAI method that helps users interpret ML models and visualize the relationships between variables (eg, sensor features in our study) and predicted outcomes (ie, BDEs, non-drinking events, and low-risk drinking events). It is based on the Shapley values from cooperative game theory [53,54] and applied in ML problems [51]. SHAP assigns contributions to features for their impact on the prediction class. In brief, (1) a greater SHAP value means greater contribution to the model prediction of a specified class, (2) a positive SHAP value means that the feature contributed “positively” to the model’s specified prediction class (increased the probability), and (3) a negative SHAP value means that the feature contributed negatively (decreased the probability) to the prediction. In the *Results* section, we present the SHAP feature importance bar plot to show the top features for BDE classification, SHAP summary plot to show how features impact BDE prediction based on the values that they take, and the SHAP dependence plots to show some of the features’ interaction with time of day and their impact on the BDE prediction on a more detailed level.

Second, we selected PDPs [52], which depict the marginal impact of 1 or 2 features on the target prediction. PDPs can show the relationship between the selected set (eg, *a test data set*) of features and their impact on drinking predictions [55]. In this study, we used PDPs to show the impact of key features (eg, radius of gyration) on the prediction of each model class and the impacts of the interaction of latitude and longitude features (ie, locations where young adults spent time) on the prediction of BDEs on weekends and weekdays using a contour plot for a circumscribed area of Pittsburgh, Pennsylvania, where the data were collected.

## Results

### Model Performance: Analysis Window Size

Overall, we found that the XGBoost models generally outperformed the logistic regression, support vector machine, random forest, and decision tree models for a range of analysis window sizes. In Table 4, we report the models with a 1-hour prediction distance and different amounts of phone sensor data (1-, 3-, 6-9-, and 12-hour windows) for weekdays. By comparing the phone sensor data with different time lengths for the same prediction distance, we found that 9 hours of phone sensor data achieved the best model. The best population model resulted in an average accuracy of 93.9% (compared with 60.6% for the baseline model that used only the day of the week feature) in predicting 3 different events during weekdays: non-drinking, low-risk drinking, and BDEs (average accuracy, *F*_1_-score, and *κ*: 93.9%, 0.94, and 0.84, respectively).

For the weekend prediction model (Table 5), we found that 12 hours of phone sensor data yielded the best model by comparing phone sensor data using different analysis window sizes (1-, 3-, 6-, 9-, and 12-hour windows) for the same predicted distance (ie, 1 hour in advance of BDE). The best population model resulted in 90.2% average accuracy (compared with 60.6% for the baseline model that used only the day of the week feature) in predicting 3 different events: non-drinking events, low-risk drinking events, and BDEs (average accuracy, *F*_1_-score, *κ*: 90.2%, 0.90, and 0.83, respectively).

**Figure 3:**
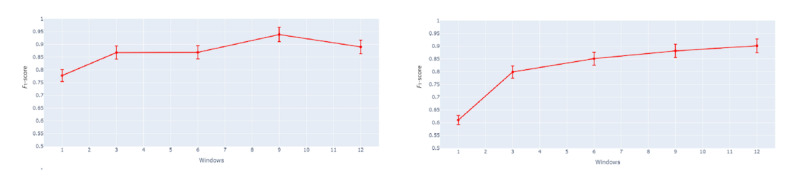
Performance comparison (F1-score) of models with different window sizes for weekdays (left) and for weekends (right).

The *F*_1_-scores for window sizes 1 to 12 hours for the weekdays (analysis distance size is held at 1 hour) are shown in Figure 3 (left). The *F*_1_-score increases according to the increase in window size until it reaches the peak when the window size is 9. When the vertex is reached, the *F*_1_-score decreases with increase in window size. WDXGBoost-W9D1 refers to the weekday XGBoost model in which the analysis window size is 9 hours and prediction distance is 1 hour from drinking onset.

The *F*_1_-scores for window sizes 1 to 12 hours for the weekends (analysis distance size is held constant at 1 hour) are shown in Figure 3 (right). The *F*_1_-score increases according to the increase in window size. WEXGBoost-W12D1 refers to the weekend XGBoost model in which the analysis window size is 12 hours and prediction distance is 1 hour from drinking onset.

**Table 4:** Performance of the models with different “analysis window” (the amount of sensor data analyzed) in predicting events, low-risk drinking events, and binge-drinking events (BDEs) 1 hour in advance during weekdays.

	Model				
	WDXGBoost-W1D1^a^	WDXGBoost-W3D1	WDXGBoost-W6D1	WDXGBoost-W9D1	WDXGBoost-W12D1
Accuracy (%)	77.8	86.8	86.9	93.9	89.0
*F*_1_-score	0.78	0.87	0.87	0.94	0.89
*κ*	0.27	0.68	0.68	0.84	0.75
BDE precision	0.33	*0.95*	0.91	0.92	0.91
BDE recall	0.33	0.69	0.68	0.84	0.73
BDE *F*_1_-score	0.33	0.80	0.78	0.88	0.81

^a^WDXGBoost-W1D1: weekday (WD) extreme gradient boosting (XGBoost) model, where analysis window size (W)=1 hour and prediction distance (D)=1 hour from drinking onset.

**Table 5:** Performance of the models with different “analysis window” (the amount of sensor data analyzed) in predicting non-drinking events, low-risk drinking events, and binge-drinking events (BDEs) 1 hour in advance during weekends.

	Model				
	WEXGBoost-W1D1^a^	WEXGBoost-W3D1	WEXGBoost-W6D1	WEXGBoost-W9D1	WEXGBoost-W12D1
Accuracy (%)	61	79.9	85.1	88.2	90.2
*F*_1_-score	0.61	0.80	0.85	0.88	0.90
*κ*	0.31	0.61	0.74	0.78	0.83
BDE precision	0.88	0.93	0.93	0.92	0.91
BDE recall	0.39	0.77	0.71	0.80	0.85
BDE *F*_1_-score	0.54	0.84	0.81	0.86	0.88

^a^WEXGBoost-W1D1: weekend (WE) extreme gradient boosting (XGBoost) model, where analysis window size (W)=1 hour and prediction distance (D)=1 hour from drinking onset.

### Model Performance: Prediction Distances From the Onset of Drinking Events

Next, we present the performance of different prediction distances, holding the analysis window size constant (weekday=9 hours and weekend=12 hours). In this section, we present the performance of the models with a range of prediction distances: 1, 3, and 6 hours from drinking events.

For the weekday prediction model, we found that the XGBoost model with a 9-hour analysis window and 6-hour prediction distance (ie, WDXGBoost-W9D6) had the highest *F*_1_-score for predicting BDEs (precision, recall, and *F*_1_-score: 0.92, 0.84, and 0.88, respectively; Table 6).

**Figure 4:**
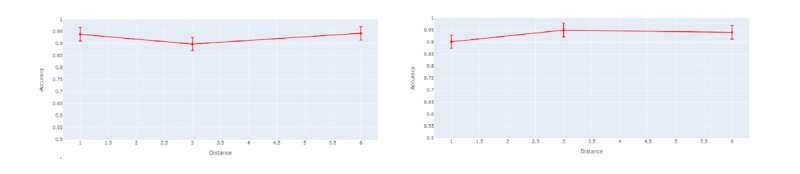
Performance comparison (accuracy) of models with different prediction distances for weekdays (left) and for weekends (right).

Figure 4 (left) shows, the model accuracy scores for prediction distances 1 to 6 hours for the weekdays (analysis window size is held constant at 9 hours). The *F*_1_-score increases according to the increase in distance until it reaches the peak when the distance is 6 hours from drinking onset. WDXGBoost-W12D1 refers to the weekday XGBoost model in which the analysis window size is 9 hours and prediction distance is 6 hours from drinking onset.

For the weekend prediction model, the XGBoost model with a 12-hour analysis window and 3-hour prediction distance (ie, WEXGBoost-W12D3) had the highest *F*_1_-score for predicting BDEs (precision, recall, and *F*_1_-score: 0.97, 0.95, and 0.96, respectively; Table 7).

Figure 4 (right) shows the model accuracy scores for prediction distances 1 to 6 hours for the weekends (analysis window size is held constant at 12 hours). The *F*_1_-score increases according to the increase in distance until it reaches the peak when the distance is 3 hours from drinking onset. “WEXGBoost-W12D3” refers to the weekend XGBoost model in which the analysis window size is 12 hours and prediction distance is 3 hours from drinking onset.

**Table 6:** Performance of models with different “prediction distances” (1-6 hours before) in predicting binge-drinking events (BDEs), non-drinking events, and low-risk drinking events during weekdays^a^.

	Model		
	WDXGBoost-W9D1^b^	WDXGBoost-W9D3	WDXGBoost-W9D6^b^
Accuracy (%)	93.9	89.8	94.3
*F*_1_-score	0.94	0.90	0.94
*κ*	0.84	0.76	0.88
BDE precision	0.92	0.92	0.92
BDE recall	0.84	0.70	0.84
BDE *F*_1_-score	0.88	0.79	0.88

^a^Analysis window size was held constant at 9 hours, which provided the best performance.

^b^WDXGBoost-W9D1: weekday (WD) extreme gradient boosting (XGBoost) model, where analysis window size (W)=9 hours and prediction distance (D)=1 hour from drinking onset.

**Table 7:** Performance of models with different “prediction distances” (1-6 hours before drinking onset) in predicting binge-drinking event (BDEs), non-drinking events, and low-risk drinking events during weekends^a^.

	Model		
	WEXGBoost-W12D1^b^	WEXGBoost-W12D3	WEXGBoost-W12D6
Accuracy (%)	90.2	95	94.1
*F*_1_-score	0.90	0.95	0.94
*κ*	0.83	0.92	0.89
BDE precision	0.91	0.97	0.92
BDE recall	0.85	0.95	0.96
BDE *F*_1_-score	0.88	0.96	0.94

^a^Analysis window size was held constant at 12 hours, which provided the best performance.

^b^WEXGBoost-W12D1: weekend (WE) extreme gradient boosting (XGBoost) model, where analysis window size (W)=12 hours and prediction distance (D)=1 hour from drinking onset.

### Predicting BDEs

In summary, the combination of the 9-hour and 12-hour analysis window sizes with a 6-hour and 3-hour prediction distances resulted in population models with the highest *F*_1_-score and accuracy for the prediction of drinking events overall on weekdays and weekends, respectively.

In addition, we were the most interested in a model that performed the best in predicting BDEs, which are associated with the greatest negative consequences. Therefore, we chose a model and hyperparameters that had the highest *F*_1_-score (balance of precision and recall) for BDEs (vs low-risk drinking and non-drinking periods) on the validation set. This same model, which had the highest *F*_1_-score, also had the highest accuracy. Specifically, the weekday model had an *F*_1_-score (for BDE) of 0.94 on the test data set and a high average precision (N, D, and BDE: 0.90, 0.96, and 0.92, respectively), recall (N, D, and BDE: 0.85, 0.99, and 0.84, respectively), and *F*_1_-score (N, D, and BDE: 0.87, 0.84, and 0.88, respectively). By contrast, the weekend model had an *F*_1_-score (for BDE) of 0.95 on the test data set and a high average precision (N, D, and BDE: 0.96, 0.91, and 0.97, respectively), recall (N, D, and BDE: 0.97, 0.90, and 0.95, respectively), and *F*_1_-score (N, D, and BDE: 0.96, 0.90, and 0.96, respectively; Tables 4-7).

### Understanding Key Contributing Features Using XAI

#### Overview

**Figure 5:**
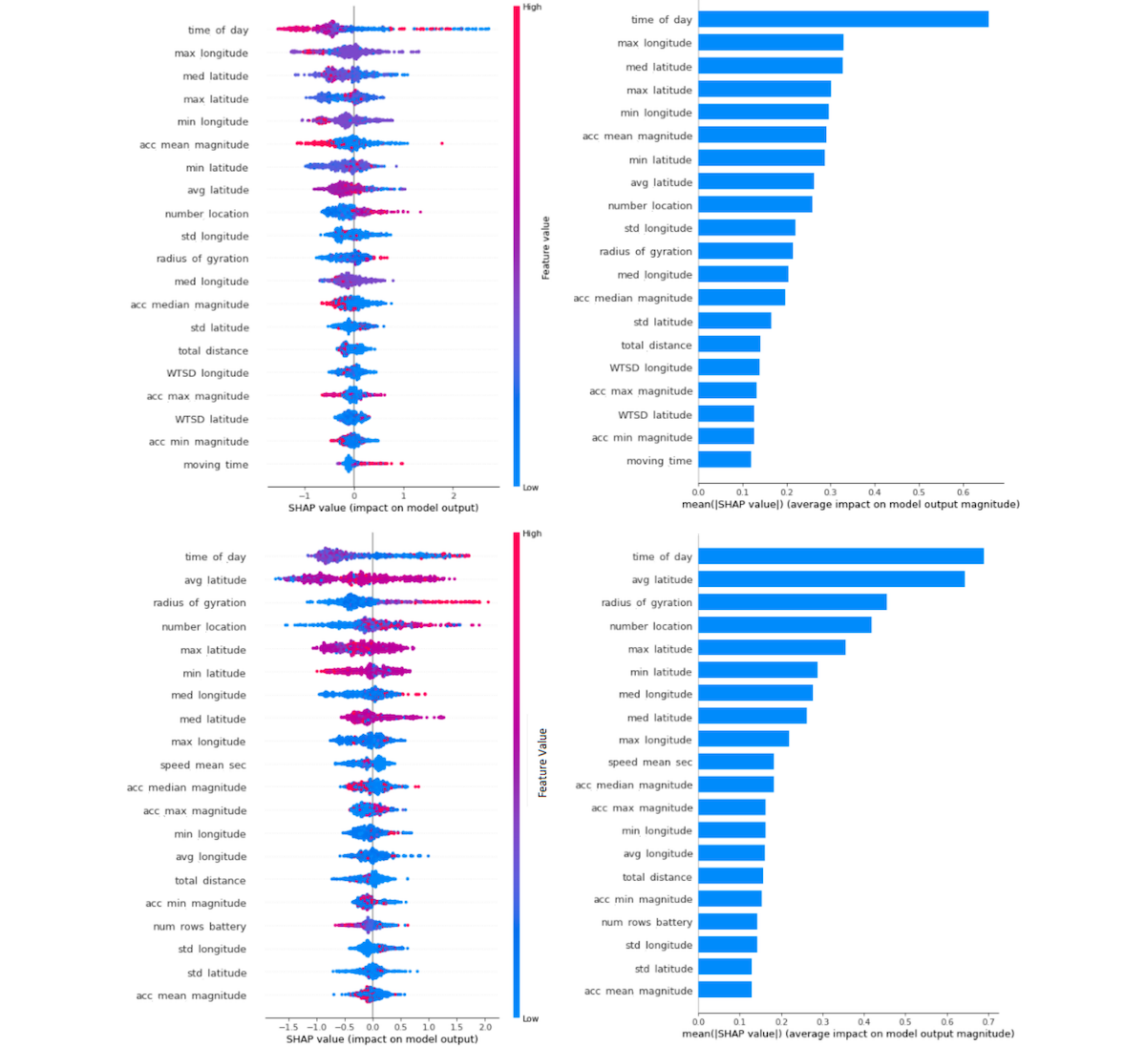
Shapley additive explanations (SHAP) summary plots show features' contribution to the models for weekdays (top) and for weekends (bottom). acc: accelerometer; avg: average; max: maximum; med: median; num: number; sec: second; std: standard deviation; WTSD: Weighted stationary latitude and longitude standard deviation.

To support JITAI development, understanding key contributors to the prediction of BDEs is needed to refine and adapt algorithm-based decisions. The SHAP feature importance plot in Figure 5 shows the 20 (default, but configurable) most important features according to their level of contributions (mean absolute SHAP values across all the instances) to the ML prediction model, only targeting BDE prediction. The results show that “time of day” contributed the most to predicting BDEs on both weekends and weekdays.

In Figure 5, we present the SHAP summary plots for our best models for both weekdays and weekends, which show the contribution of sensor features to the prediction of BDEs (left) and feature importance (right) on weekdays (top) and on weekends (bottom) using the test data set. SHAP summary plots show each sample in the test set as a data point and their impact in predicting BDEs based on the relative values of features. The color spectrum depicts the values of features. If the value of a feature is relatively high for a specific instance, it is represented in red. If the value is relatively low, it is represented in blue. The plots on the right-side show feature contribution in absolute SHAP values for the most influential 20 features.

Overall, *the longitudinal and latitudinal coordinate* statistics, *radius of gyration*, and *movement* features were among the most highly influential features for BDE prediction. SHAP results show that (1) latitude and longitude were the most important features, in addition to time of day contributing to the prediction of BDEs both on weekdays and weekends, and (2) radius of gyration is a relatively more impactful predictor of BDEs on weekends than on weekdays.

From the summary plots on the left, we can see that acceleration features tend to be negatively related to the BDE prediction on both the weekends and weekdays, meaning that higher acceleration values decrease the probability of BDE prediction. This greater activity or movement of the smartphone is associated with a lower likelihood of BDEs. In addition, radius of gyration and number of locations are positively related to the BDE prediction (ie, greater radius of gyration and number of locations increase the probability of BDE prediction).

#### SHAP: Interaction Between Time of Day and Radius of Gyration

**Figure 6:**
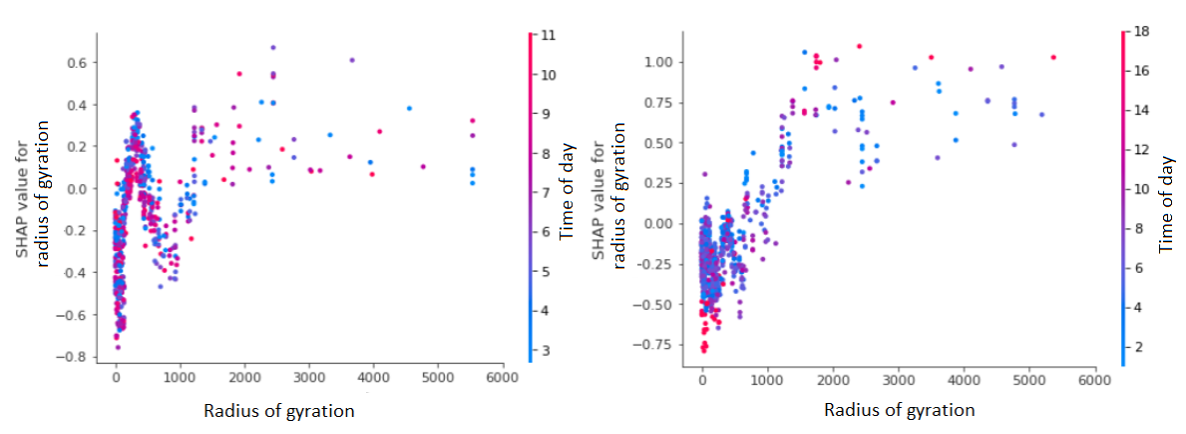
Shapley additive explanations (SHAP) dependence plots present an interaction between the time of day and the radius of gyration for weekdays (left) and for weekends (right).

We present the interaction effect between key features: time of day with GPS-derived travel pattern or location. As shown in Figure 6, the results demonstrate the interaction between the radius of gyration and time of day features and their impact on BDE prediction. The hours of the day are represented with colors, and the SHAP value represents the impact on the BDE prediction—positive SHAP value indicates a positive contribution to BDE prediction.

The radius of gyration followed similar patterns on both weekends and weekdays for values greater than approximately 1000 m; however, radius of gyration and day of week (weekday or weekend) had opposite patterns for values between 0 and 800 m contributing to BDEs on weekdays. When radius of gyration is between 0 and 800 m, SHAP values for weekdays follow an inverted u–curve peaking at around 500 m, whereas for weekends, the same radius of gyration range follows a u-curve (Figure 6 left [weekdays] vs right [weekends]).

In addition, we narrowed down the coordinates to solely represent the Pittsburgh area (ie, the place where data were collected) to localize the meaning of the values. Looking at the average latitude plots, we see that the patterns are inverse of each other for weekdays and weekends for the same latitudinal coordinates, which means that young adults visit certain areas before BDEs on the weekends but not on weekdays. This is highly likely owing to bars and other drinking spaces being in the vicinity of workplaces in the city.

#### PDP: Contribution of the Features to the Prediction of BDEs Versus non-drinking and Low-Risk Drinking Events

**Figure 7:**
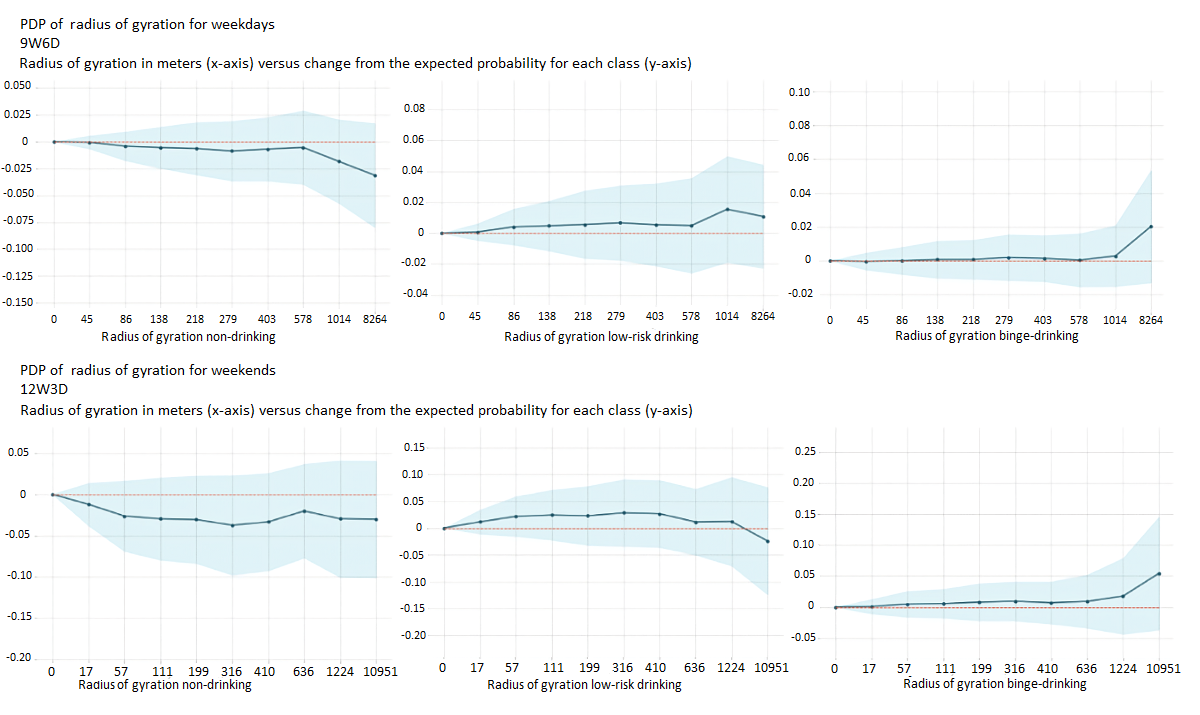
Two-way partial dependence plots (PDPs) on the effects of latitude and longitude on binge-drinking event (BDE) probability. 9W6D: the model by taking 9-hour window and 6-hour analysis distance from the onset of BDE; 12W3D: the model by taking 12-hour window and 3-hour analysis distance from the onset of BDE.

PDPs show the marginal effect that 1 or 2 features have on the ML model [52]. In Figure 7, we visualize the impact of radius of gyration (which refers to the radius of the circle that includes all locations visited during a time window) on each prediction class.0 on the y-axis represents the expected (mean) probability value of each respective class. Positive y-axis values indicate positive contribution to the prediction of the class, whereas the negative y-axis values indicate negative contribution to the prediction of that class. x-axis values are radius of gyration values in meters, divided into 9 equal portions (ie, the number of samples between grids are equal).

From the plots, we can see that young adults are more likely to travel within a larger area (radius of gyration) before BDEs compared with non-drinking or low-risk drinking events on weekdays. By contrast, it is shown that a greater radius of gyration on weekends is negatively related to non-drinking positively related to low-risk drinking events and BDEs. However, specifically the range between 1223 and 10,952 m was positively related to BDEs. Furthermore, on weekends, the probability of not drinking alcohol declines as the radius of gyration increases, whereas on weekdays, it follows a more linear pattern with a slight increase in probability at higher radius of gyration.

#### Two-Way PDPs for Location Coordinates

**Figure 8:**
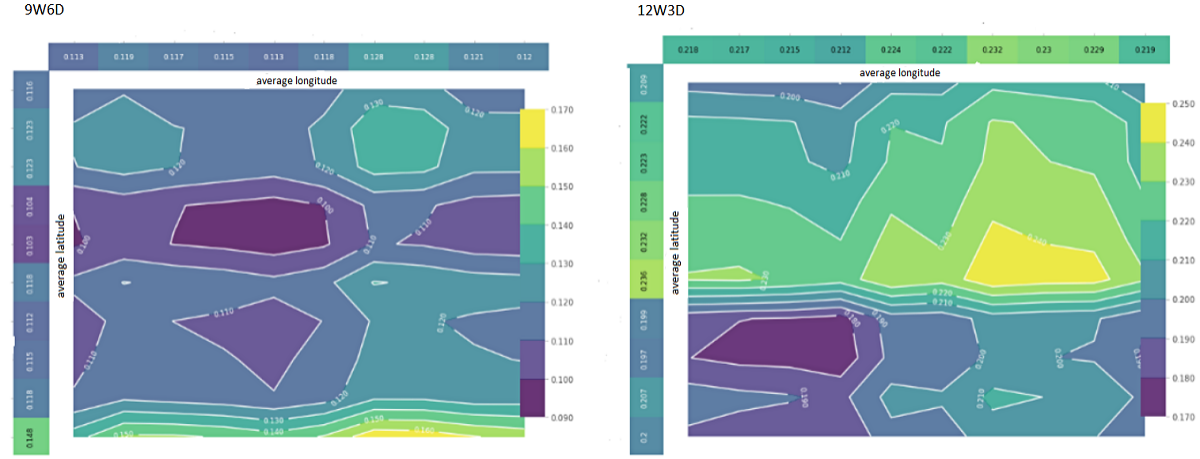
Shapley additive explanations (SHAP) summary plots show features' contribution to the models. (A) PDP interaction of location coordinates for weekdays. (B) PDP interaction of location coordinates for weekends. PDP: Partial Dependence Plots.

In Figure 8, we present the PDP of BDE on weekdays (left) and weekends (right) and the interaction of 15-minute average longitudes and latitudes on a contour plot representing a section in the Pittsburgh metropolitan area (city of Pittsburgh and surrounding smaller towns). 9W6D refers to the model by taking 9-hour window and 6-hour analysis distance from the onset of BDE and 12W3D refers to the model by taking 12-hour window and 3-hour analysis distance from the onset of BDE.

This plot can be used as a map to display which visited areas increase the probability of BDEs on weekends versus weekdays. The horizontal axes represent the average latitude values, and the vertical axes represent the average longitude values. To preserve privacy and simplify the plot, rather than mapping out the exact locations of participants and the probability of BDEs at specific locations, we plotted their general locations (zip code level or neighborhood level) to give a bird’s eye view on their locations and calculate the probabilities in general areas rather than specific locations. The color spectrum represents the probability of the person starting a BDE within 6 to 15 hours later that day. From dark to bright colors, the probability of BDEs increases.

From the weekend plot, we found that in certain geographic locations (represented in yellow in the plot), the probability of BDEs was 24%, higher than the expected probability of 21.4% (relative increase of 11% and absolute increase of 2.6%).

As for the weekday plot, in the areas colored in yellow, the average probability of binge drinking behavior was 17%, higher than the expected probability of BDE of 11.9% in the entire data set, which indicates a 1.43× increase in BDE prevalence (weekday plot analyzes a greater area because of the differences in how the data are dispersed). Thus, it can be concluded that just by looking at the 15-minute average locations of young adults who reported hazardous alcohol use and were sampled from an ED in the Pittsburgh metropolitan area, we can make meaningful inferences about their probability of binge drinking later that day. With more fine-grained and personalized GPS data, it may be possible to increase the predictive power of location information.

In summary, we found that a combination of time of day and GPS-derived travel and location features provided interpretable patterns of young adults who are likely to report same-day BDEs. Our findings not only optimized the ML model with “windows of opportunity” but also identified “key location features” to trigger JITAIs to support the design of intervention strategies and messages for BDE prevention among young adults.

## Discussion

### Principal Findings

#### Overview

We attempted to predict imminent same-day BDEs in young adults with the use of smartphone-based sensors. We used ML coupled with XAI *to investigate windows of opportunity to prevent BDEs.* Our results confirm that smartphone sensors can be used to predict imminent BDE events before their onset using same-day behaviors and comparing them with the behaviors during non-drinking and low-risk drinking events on weekdays and weekends. The key contributing features we found are time of day, travel patterns and boundaries (eg, radius of gyration), and accelerations of body movements, which could be used for triggering JITAI before the onset of BDEs and thus preventing the potential risks of BDEs.

As our previous work confirmed that the *detection* of BDEs is feasible using phone sensor data [17,18], *moving toward the prevention of BDEs,* we focused on predicting imminent (same-day) BDEs before their onset. In this feasibility study, we demonstrated how to build an ML model using passively sensed smartphone sensor data, which predicted young adult drinking behaviors (whether they will engage in BDEs, low-risk drinking events, or non-drinking events), with 94.3% accuracy for weekday drinking behavior (WDXGBoost-W9D6) and 95% accuracy for weekend drinking behavior (WEXGBoost-W12D3). The prediction model using phone sensor data for both weekday and weekend drinking had higher accuracy relative to a baseline model using only day of the week to predict drinking behavior (60.6% accuracy). We also found that 9 hours (for weekdays) and 12 hours (for weekends) of phone sensor data collected at a 6-hour and 3-hour prediction distance (weekday and weekend, respectively) maximized the *F*_1_-score (which balances precision and recall) for predicting BDEs. The analysis window size is important for estimating data storage needs and potential privacy risks of storing sensitive data on the phone for prediction. This ML prediction model advances our prior work using phone sensors to “detect” episodes of alcohol use that have already started [17,18] by allowing the delivery of JIT support messages 3 and 6 hours before a predicted drinking event, when alternative plans to support drinking limit goals can be considered and implemented.

#### Important Smartphone Sensor Features for Predicting BDEs

There were similarities in the most important phone sensor features for the “detection” and “prediction” of drinking events. For example, time (eg, time of day and day of the week) was important for both the “detection” [17,18] and prediction of drinking events. However, there were important differences in key features used to “detect” relative to “predict” a drinking event. As an example, microlevel features, such as screen interaction duration and number of activity changes, were key features relevant to the “detection” of a drinking event, whereas the macrolevel features of travel and location (eg, radius of gyration and latitude and longitude) were key features relevant to the “prediction” of an imminent drinking event. XAI further revealed that the interaction of time (eg, time of day and day of the week) with travel and location features contributed to the prediction of drinking events in young adults.

In predicting drinking events, we found that young adults who traveled more (eg, larger radius of gyration) and *spent longer duration in important locations* were more likely to report BDEs (ie, compared with non-drinking events) and drinking events (ie, non-drinking and drinking events) *later that day.* Furthermore, the participants who interacted with their smartphones more (ie, higher acceleration values) and had longer call durations were less likely to drink alcohol later in the day. These results suggest that smartphone interaction activity and communication during the day might indicate work, school, and social obligations and activities that could help to regulate or constrain drinking behavior. By contrast, young adults who had *fewer communication* interactions using the phone had an increased likelihood of drinking later that day. The low level of communication activity involving the phone in the hours before a drinking event may reflect a sense of social isolation, which is a direction to be examined in future research. The most important smartphone sensor features contributing to drinking behavior prediction may serve as early warning signals, which have parallels in the mental health literature, where, for example, certain signs and symptoms signal increases in depressive symptom severity [56].

Further analyses using XAI indicated differences in the importance of features in predicting BDEs in young adults on weekdays relative to weekends. For example, the radius of gyration was a more important predictor of BDEs on weekends than on weekdays. The relevance of GPS-derived travel data in relation to episodes of alcohol use is consistent with prior smartphone sensor work with young adults [16]. Our use of XAI adds to this emerging literature by showing, for the first time, that the interaction of day of the week, time of day, and location information contributed to the prediction of BDEs in young adults. The enhanced explainability of the ML prediction model provides novel insight into factors that serve as early warning signs of an imminent and potentially preventable BDE in young adults.

#### JIT Delivery to Prevent BDEs

The ability to predict upcoming drinking events 3 and 6 hours before their likely onset during weekends and weekdays, respectively, using only smartphone sensor data could support JIT intervention [7], potentially boosting digital intervention effects [57]. This time window prior to drinking onset permits the opportunity for a more proximally timed, proactive intervention in young adult drinkers to modify their drinking intentions, enhance their motivation to set drinking limit goals, and provide them with tips to successfully meet their health goals when such support may be most salient. In several studies, including Suffoletto et al [35], we found that proactively prompting goal commitment to limit drinking before drinking onset is a critical component of JIT interventions. As such, a prediction-based intervention approach avoids delivering messages or support during a drinking event when cognitive and motivational impairment due to acute alcohol use might limit message utility.

The drinking prediction models presented in this paper, testing a range of distances from the onset of drinking (eg, 1-, 3-, and 6-hour prediction distances), suggest temporal changes that are the most predictive of drinking on specific days (weekends vs weekdays), which are relevant to tailoring intervention content [7]—for example, *farther from the onset of drinking*, *enhancing the motivation to limit drinking*, *or planning alternative healthy activities might be prioritized*. In contrast, once drinking starts (ie, relevant to a drinking detection model), specific techniques to reduce the risk associated with a BDE could be provided (eg, alternate alcoholic and nonalcoholic drinks) [4]. We envision the future use of our predictive models to prevent the onset of BDEs and suggest some considerations to developers that need to be considered for designing JIT intervention systems. In this system, young adults willingly download an app on their smartphone, consent to the analysis of phone sensor data, and receive intervention messages to avoid BDEs and related negative consequences. To make the app relevant and useful to a young adult, JIT resources could be sent to promote rewarding, personalized nonalcohol events that shift attention and intention away from drinking to healthy alternative activities.

#### Privacy and Data Storage

Our results suggest that an eventual BDE prediction and intervention app requires a certain amount of sensor data to be temporarily stored on one’s smartphone. The phone needs to store a window of 12 hours (WEXGBoost-W12D3) and 9 hours (WDXGBoost-W9D6) of data to run the prediction algorithm for BDEs on weekends and weekdays, respectively. Older data can be removed on an ongoing basis to maximize data privacy. Furthermore, although our current system runs on a server, the prediction model is efficient enough that it could be executed directly on a smartphone, with data never leaving the smartphone. Regarding privacy and ethical issues in the collection of phone sensor data, there is a need to carefully review the data collection process with the participants, including the collection of specific types of sensor data (eg, location data) and the granularity at which data will be collected (ie, the precision of location data to minimize identifiability), and provide them with examples of the variables that will be derived (eg, the radius of daily travel from location data) so that they can evaluate the potential risks to privacy and provide informed consent for data collection. The voluntariness of data collection and ability to opt out of data collection at any time need to be made clear and discussed with the participants when obtaining their informed consent. The project’s app used reminders (eg, flashing red banner or notification saying that the app was running) to keep the participants informed of phone sensor data collection. Secure methods of data transmission and storage (eg, on the phone and server) were used but are subject to potential breach, a risk which the participants need to understand.

#### Privacy, Trust, and XAI

It is challenging to balance privacy preservation and the accuracy of knowing an individual’s location and travel patterns. The collection and analysis of GPS data raise privacy issues because the participant’s location (eg, home address) might be identifiable. The deidentification and level of granularity of data must be considered in protecting the privacy and confidentiality of the participants. Interestingly, based on our supplementary analysis (details in Multimedia Appendix 1) using the rounded GPS latitude and longitude coordinates, we found a trade-off relationship between privacy and accuracy. The supplementary experiment showed that accuracy was 5 percentage points lower compared with our best performing model (94%) when the latitude and longitude coordinates were rounded (to 1 decimal place; ie, 0.1 refers to approximately 11.1 km). In this scenario (GPS data rounded to 1 decimal place), GPS data do not represent the specific address that the participants visit during the day but instead represent a relatively large region, ranging from 10 to 50 km. As such, it is important to consider the granularity of GPS data to make meaningful BDE predictions that balance privacy-preserving strategies and the performance of the prediction algorithms. Alternatively, after a full explanation of the risks and data to be collected, the participants may agree to the collection and analysis of granular location data for research and health care purposes. These location data, along with analysis of key behavioral features captured by smartphone-based sensors impacting BDEs, permit the use of XAI techniques, including feature importance and contribution (via SHAP and PDPs) to determine potential features contributing to the prediction of imminent BDEs. In addition, XAI models that explore counterfactuals (eg, non-BDEs) and contrastive examples (eg, low-risk drinking) [58,59] may be used to understand hypothetical alternative scenarios and generate possible feature combinations to obtain an expected outcome (eg, non-drinking). However, risks such as overtrust and reliance [60] on clinician decision support systems need to be carefully investigated.

#### Real-world Application and Related Issues

More frequent reminder systems or a more effective incentive mechanism could improve compliance with sustainable data collection using phone sensors and phone surveys over a long period. The development of a “dashboard” could help a researcher to monitor participant compliance. Alternatively, newer transdermal alcohol biosensors might facilitate the collection of data on alcohol consumption with less stigma and participant burden for use in providing “ground truth” to train the ML model.

Regarding battery drain, future research on the use of smartphone sensors for predicting drinking events might identify an optimal set of sensors to use. This optimal set could use a lower overall sampling rate or optimize the sampling rate for the sensors based on the algorithm chosen to minimize battery drain. Although we used the default sampling rates specified in the AWARE data collection framework, in the future, we can explore different sampling rates to optimize both the performance of the predictive models and the battery life of the smartphones. Future studies should identify which phone sensor features are acceptable to participants for monitoring purposes to allow for more personalized support.

### Limitations and Future Work

Although our initial results are promising, there are some limitations that should be mentioned. First, the participants self-reported alcohol consumption and the timing of starting and stopping drinking, which might result in recall biases because of the retrospective nature of self-reports. Second, phone sensors, although useful, cannot capture and infer all relevant behaviors associated with a drinking event. Third, some aspects of drinking event planning are not manifested in outward behaviors (eg, subjective craving). Fourth, we successfully built a population model to predict non-drinking events, low-risk drinking events, and BDEs in young adults; however, there might be individual patterns that our population model cannot capture, which may limit the generalizability of our model. In future work, we will collect a larger data set per participant with the integration of other phone data (eg, social media posts) and investigate the use of more personalized individual models. Our analyses focused on calls (incoming or outgoing) and SMS text messages as the main form of communication rather than communication through social media apps. Personalized models could result in a more accurate prediction system at the individual level.

As is the case for any predictive model, deployment will result in some false positive and false negative predictions of drinking or BDEs. The potential “cost” of a false positive is that an intervention may not only be delivered without need, bothering an individual when irrelevant and degrading credibility, but also may lead someone to consider drinking when they did not originally intend to drink. Alternatively, not delivering an intervention when an opportunity to do so occurs (false negative) could result in perceived program unreliability; therefore, this should also be taken into account when selecting the optimal model.

The studied sample comprised young adults who screened positive for hazardous drinking and were recruited from an urban area in western Pennsylvania. As such, the findings may not be directly applicable to other age groups, those with lower levels of drinking, and those from rural areas with different activity patterns.

We position our work as a feasibility study, as our study population was limited to young adults. As we would expect that both the phone use behaviors and the drinking behaviors of young adults will differ from those of older adults, we do not claim that our approach or results will be generalizable to a population with different demographics. Instead, we suggest that future work needs to replicate the data collection and analyses reported here on a study population with a wider range of demographics. This replication will determine how generalizable our results are, both with a larger and more heterogeneous young adult population and with an older adult population.

The XAI-integrated BDE model we developed could allow clinicians to assist in designing JITAIs for preventing imminent BDEs and thus their potential negative consequences. The specific sensor features contributing to high model performance highlight the potential for interpreting young adults’ behaviors only using smartphones in an unobtrusive way. As such, it should enable clinicians to derive advanced strategies for JITAI for young adults to prevent the onset of a BDE.

### Conclusions

To the best of our knowledge, this is the first study to attempt to develop a prediction model of same-day imminent BDE events using smartphone sensor data with a maximum accuracy of 95% (weekend model), which outperformed a baseline model that used only day of the week to predict same-day drinking in young adults. The best-performing prediction model was the XGBoost, using a 9-hour and 12-hour analysis window size at a prediction distance of 6 and 3 hours before the onset of a BDE on weekdays and weekend, respectively. To improve interpretability of the BDE model, we applied XAI (eg, SHAP and PDPs) to determine key features and their contributions. The “windows of opportunity” and the key contributors (eg, the time of day, radius of gyration, and acceleration of movement) can be used by clinicians for designing JIT messaging to prevent imminent BDEs in young adults.

## Appendix

Multimedia Appendix 1 Supplementary materials.

## Abbreviations

BDE: binge-drinking event
D: low-risk drinking event
ED: emergency department
EMA: ecological momentary assessment
iOS: iPhone operating system
JIT: just-in-time
JITAI: just-in-time adaptive intervention
N: non-drinking event
PDP: partial dependence plot
SHAP: Shapley additive explanations
SMOTE: synthetic minority over-sampling technique
XAI: explainable artificial intelligence
XGBoost: extreme gradient boosting

## Data Availability

The GitHub repository for the study [61] is open to the public. To preserve privacy, we rounded the latitude and longitude coordinates to 1 decimal place, that is, 0.1 refers to approximately 11.1 km distance, in the data set. Retaining the raw location data might cause potential risks to participants’ privacy, as participants’ home addresses would be identifiable. High-level location features such as the radius of gyration are available to access in the data set.
