# Supplementary material for: Leveraging Mobile Phone Sensors, Machine Learning, and Explainable Artificial Intelligence to Predict Imminent Same-Day Binge-drinking Events to Support Just-in-time Adaptive Interventions: Algorithm Development and Validation Study
Source: JMIR Form Res. 2023 May 4;7:e39862. doi: 10.2196/39862 (PMC10196900; doi:10.2196/39862)
Supplement: Multimedia Appendix 1 [file formative_v7i1e39862_app1.docx]

**Supplementary Materials**

1. **Phone sensor features used in the analysis**

| No.Order in the dataset | name | Category |
| --- | --- | --- |
| 1 | acc min magnitude | Movement |
| 2 | acc max magnitude | Movement |
| 3 | acc mean magnitude | Movement |
| 4 | acc median magnitude | Movement |
| 5 | acc std magnitude | Movement |
| 6 | length of charge minutes | Battery |
| 7 | num rows battery | Battery |
| 8 | avg call duration | Device usage |
| 9 | std call duration | Device usage |
| 10 | min call duration | Device usage |
| 11 | max call duration | Device usage |
| 12 | total call duration | Device usage |
| 13 | number rows calls | Device usage |
| 14 | number incoming calls | Device usage |
| 15 | number missed calls | Device usage |
| 16 | number of correspondents phone | Device usage |
| 17 | number outgoing calls | Device usage |
| 18 | number rows messages | Device usage |
| 19 | number of outgoing messages | Device usage |
| 20 | number of incoming messages | Device usage |
| 21 | number of correspondents | Device usage |
| 22 | location variance | Location |
| 23 | location variance log | Location |
| 24 | total distance meters | Location |
| 25 | speed mean meters per sec | Location |
| 26 | speed var meters per sec | Location |
| 27 | location entropy | Location |
| 28 | location entropy normalized | Location |
| 29 | max len stay at clusters in minutes | Location |
| 30 | mean len stay at clusters in minutes | Location |
| 31 | min len stay at clusters in minutes | Location |
| 32 | std len stay at clusters in minutes | Location |
| 33 | moving time percent | Location |
| 34 | number location transitions | Location |
| 35 | radius of gyration | Location |
| 36 | number of clusters | Location |
| 37 | time at cluster 1 | Location |
| 38 | time at cluster 1 in group | Location |
| 39 | time at cluster 2 | Location |
| 40 | time at cluster 2 in group | Location |
| 41 | time at cluster 3 | Location |
| 42 | time at cluster 3 in group | Location |
| 43 | unlocks per minute | Device usage |
| 44 | number of minutes interaction | Device usage |
| 45 | number of minutes unlock | Device usage |
| 46 | max len minute interaction bout | Device usage |
| 47 | min len minute interaction bout | Device usage |
| 48 | mean len minute interaction bout | Device usage |
| 49 | std len minute interaction bout | Device usage |
| 50 | max len minute unlock bout | Device usage |
| 51 | min len minute unlock bout | Device usage |
| 52 | mean len minute unlock bout | Device usage |
| 53 | std len minute unlock bout | Device usage |
| 54 | time of day | Time/Day |
| 55 | avg latitude | Location |
| 56 | avg longitude | Location |
| 57 | med latitude | Location |
| 58 | med longitude | Location |
| 59 | std latitude | Location |
| 60 | std longitude | Location |
| 61 | WTSD latitude | Location |
| 62 | WTSD longitude | Location |
| 63 | max latitude | Location |
| 64 | max longitude | Location |
| 65 | min latitude | Location |
| 66 | min longitude | Location |
| 67 | total distance | Location |
| 68 | speed mean sec | Location |
| 69 | moving time | Location |
| 70 | number location | Location |

1. **Features Description**

| Category | Features selected by Random Forest | Description &  Implementation details | Ref. |
| --- | --- | --- | --- |
| TIME | Time of day | The time at which this entry occurred (*0-23 hours*).  Calculate what time the data is located based on the timestamp | [17] |
| LOC | max latitude  med latitude  min latitude  avg latitude  std latitude  max longitude  med longitude  min longitude  avg longitude  std longitude | Statistics of latitude and longitude within the time window  1) Remove noise records, select location records  2) Calculate *maximum, minimum, median, mean, and standard deviation* | [43] |
| LOC | Radius of gyration | The radius of gyration of all valid transition locations within the time window  1) Remove noise records (*where location label==-1*), select location records where location label changes from previous records as *"transition locations"*; 2) Mathematically, the radius of gyration here is *the root mean square distance* of all transition locations from the center of them; Calculated as follows: *sqrt(((lat1-lat mean)^2+(lon1-lon mean)^2+(lat2-lat mean)^2+(lon2-lon mean)^2+......)/num transitions)* | [17] |
| LOC | number location | Locations passed by participants within the time window  1) Remove noise records, select location records 2) Calculate the duration interval between two data 3) Calculate the distance between two data 4) Calculate the speed between two data by duration and distance 5) If speed *>0.3m/s*, we identify the second data as the moving point 6) If a participant moves from the previous data as a moving point and the following data as a stationary point, we assume that the person moves to another place. *number location add 1.* | *Self-developed* |
| LOC | speed mean sec | Average speed of participants within the time window  1) Remove noise records, select location records 2) Calculate the speed  Calculated as follows:  *total distance/moving time* | [17] |
| LOC | weighted stationary latitude  weighted stationary longitude | The variation of the locations the participants visited within the time window  1) Remove noise records, select location records  2) Calculate the duration interval between two data 3) Calculate the distance between two data 4) Calculate the speed between two data by duration and distance 5) If speed *>0.3m/s*, we identify the second data as the moving point 6) To calculate standard deviation by considering the time spent at each location Calculated as follows: *sqrt(cov(latitude, duration)*  *sqrt(cov(longitude, duration)* | [43] |
| LOC | total distance | The total distance moved by the participant within the time window  1) Remove noise records, select location records  2) Calculate the duration interval between two data 3) Calculate the distance between two data 4) Calculate the speed between two data by duration and distance 5) If speed *>0.3m/s,* we identify the second data as the moving point 6) Calculate the total distance of all moving points within the time window | *Adopted by [43] and created* |
| LOC | moving time | Time for participants to move within the time window  1) Remove noise records, select location records  2) Calculate the duration interval between two data 3) Calculate the distance between two data 4) Calculate the speed between two data by duration and distance 5) If speed *>0.3m/s*, we identify the second data as the moving point 6) Calculate the total time of all moving points within the time window | *Adopted by [43] and created* |
| MOV | acc median magnitude  acc mean magnitude  acc max magnitude  acc min magnitude  acc std magnitude | The median, mean, maximum, minimum, and standard deviation phone accelerate the magnitude of the participant within the time window | [17] |
| BAT | num rows battery | The number of cell phone battery data generated within the time window | [17] |
| DEV | unlocks per minute | The number of times participants unlock their phones per minute within the time window. | [17] |
| DEV | mean len minute interaction bout  min len minute interaction bout  max len minute interaction bout | Average, minimum, and maximum interaction time between a participant and screen within the time window | [17] |
| DEV | number of minutes interaction | The total number of interactions between a participant and screen within the time window | [17] |
| DEV | mean length minute unlock bout | Average time for unlocking the phone within the time window | [17] |

*LOC: location, MOV: movement, BAT: battery usage, DEV: device usage*

*The time window refers to a 15-minutes, a unit of analysis in our study*

*Max=maximum, min=minimum, med=median, len=length, acc=acceleration, std=standard deviation, sec=second, avg=average, num=number, m=meter, s=second, sqrt=square root, cov= covariance, lat=latitude, lon=longitude*

1. **Extra Analysis with Rounded Location Data**

To protect the privacy of the participants, we do not disclose granular location data, We keep latitude and longitude data to one decimal place.

We report the models with a 1-hour prediction distance and different amounts of phone sensor data (1-, 3-, 6- 9- and 12-hour windows) for Weekday. Comparing the performance of models using phone sensor data with different amounts of phone sensor data (e.g., 1-, 3-, 6-, 9-, 12-hours) for the same prediction distance (e.g., 1-hour), we found that 9 hours of phone sensor data achieved the best model for Weekdays.

Table c-1. Performance of the models with different *“Analysis Window"* (amount of sensor data analyzed) *for Weekdays in* predicting Non-Drinking, Low-Risk Drinking and Binge Drinking Event (BDE) 1 hour in advance

| Model | WDXGBoost -W1D1 | WDXGBoost -W3D1 | WDXGBoost -W6D1 | **WDXGBoost -W9D1** | WD XGBoost -W12D1 |
| --- | --- | --- | --- | --- | --- |
| Accuracy | 66.7% | 81.1% | 85.2% | **87.4%** | 85.8% |
| F1 | 0.67 | 0.81 | 0.85 | **0.87** | 0.86 |
| Kappa | 0.15 | 0.53 | 0.65 | **0.71** | 0.69 |
| BDE Precision | 0.33 | 0.70 | 0.81 | **0.88** | 0.81 |
| BDE Recall | 0.22 | 0.73 | 0.67 | **0.71** | 0.64 |
| BDE F1 | 0.27 | 0.71 | 0.76 | **0.79** | 0.72 |


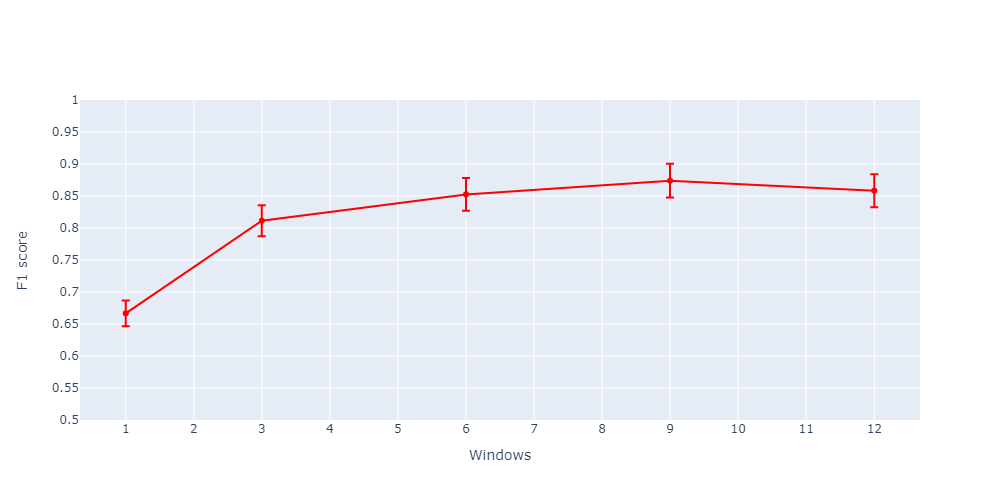


Figure c-1. WDXGBoost -W9D1 = Weekday XGBoost - Analysis Window Size = 9 hours and Prediction Distance = 1 hour from drinking onset.

We do the same for the weekend dataset, by comparing the phone sensor data with different analysis window sizes (e.g., 1-, 3-, 6-, 9-, 12-hours) for the same prediction distance (1 hour). We found that 12 hours of phone sensor data achieved the best model for Weekends.

Table c-2. Performance of the models with different *“Analysis Window"* (amount of sensor data analyzed) *for Weekend in* predicting Non-Drinking, Low-Risk Drinking and Binge Drinking (BD) Event 1 hour in advance

| Model | WEXGBoost -W1D1 | WEXGBoost -W3D1 | WEXGBoost -W6D1 | WEXGBoost -W9D1 | **WEXGBoost -W12D1** |
| --- | --- | --- | --- | --- | --- |
| Accuracy | 62.7% | 74.3% | 79.7% | 81.2% | **88.0%** |
| F1 | 0.63 | 0.74 | 0.80 | 0.81 | **0.88** |
| Kappa | 0.32 | 0.55 | 0.63 | 0.65 | **0.79** |
| BDE Precision | 0.44 | **1.0** | 0.78 | 0.79 | 0.88 |
| BDE Recall | 0.50 | 0.52 | 0.69 | 0.70 | **0.84** |
| BDE F1 | 0.47 | 0.68 | 0.73 | 0.74 | **0.86** |


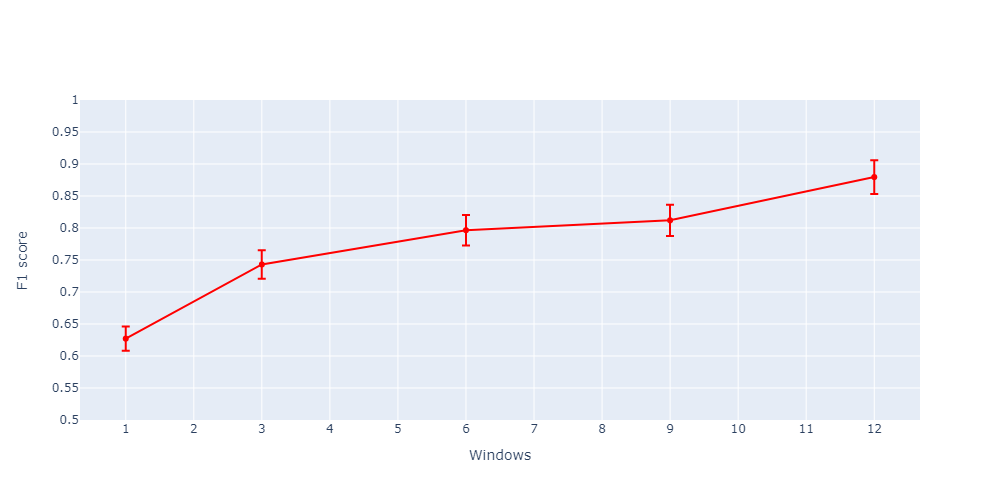


Figure c-2. WEXGBoost -W12D1 = Weekend XGBoost - Analysis Window Size = 12 hours and Prediction Distance = 1 hour from drinking onset.

For Weekdays, using the analysis window size with the best performance (9 hours), we tested models with different “prediction distances” (1-6 hours) in predicting Binge Drinking Event (BDE), Non-Drinking and Low-Risk Drinking

**Table c-3** Performance of models with different *“Prediction Distances”* (1-6 hours before) for Weekdays in Predicting Binge Drinking Event (BDE), Non-Drinking and Low-Risk Drinking

| Model | WDXGBoost -W9D1 | WDXGBoost -W9D3 | **WDXGBoost -W9D6** |
| --- | --- | --- | --- |
| Accuracy | 87.4% | 90.6% | **90.7%** |
| F1 | 0.87 | 0.91 | **0.91** |
| Kappa | 0.71 | 0.78 | **0.81** |
| BDE Precision | 0.88 | **0.92** | 0.83 |
| BDE Recall | 0.71 | 0.77 | **0.77** |
| BDE F1 | 0.79 | **0.84** | 0.80 |

For the weekday prediction model, we found that the XGBoost model with a 9-hour analysis window and 6-hour prediction distance had the highest F1-score for predicting binge drinking events


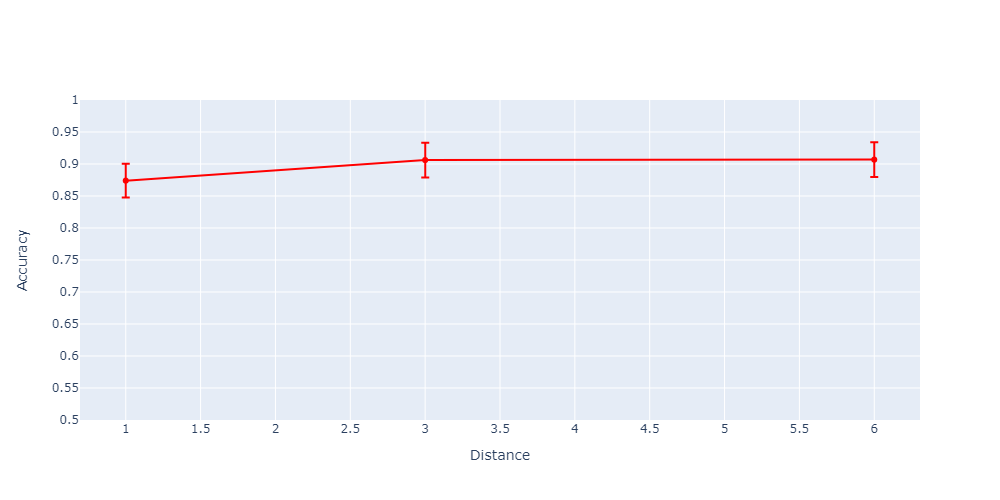


**Figure c-3.** Weekday XGBoost – Analysis Window Size = 9 hours and Prediction Distance = 6 hours from drinking onset.

We do the same for the weekend dataset, using the analysis window size with the best performance (12 hours), we tested models with different “prediction distances” (1-6 hours) in predicting Binge Drinking Event (BDE), Non-Drinking and Low-Risk Drinking

**Table c-4.** Performance of models with different *“Prediction Distances"* (1-6 hours before drinking onset) for Weekend in Predicting BDE, Non-Drinking and Low-Risk Drinking

| Model | WEXGBoost -W12D1 | WEXGBoost -W12D3 | **WEXGBoost -W12D6** |
| --- | --- | --- | --- |
| Accuracy | 88.0% | 89.9% | **91.1%** |
| F1 | 0.88 | 0.90 | **0.91** |
| Kappa | 0.79 | 0.82 | **0.84** |
| BDE Precision | 0.88 | **0.91** | 0.83 |
| BDE Recall | 0.84 | 0.86 | **0.96** |
| BDE F1 | 0.86 | 0.88 | **0.89** |

For the weekend prediction model, we found that the XGBoost model with a 12-hour analysis window and 6-hour prediction distance had the highest F1-score for predicting binge drinking events.


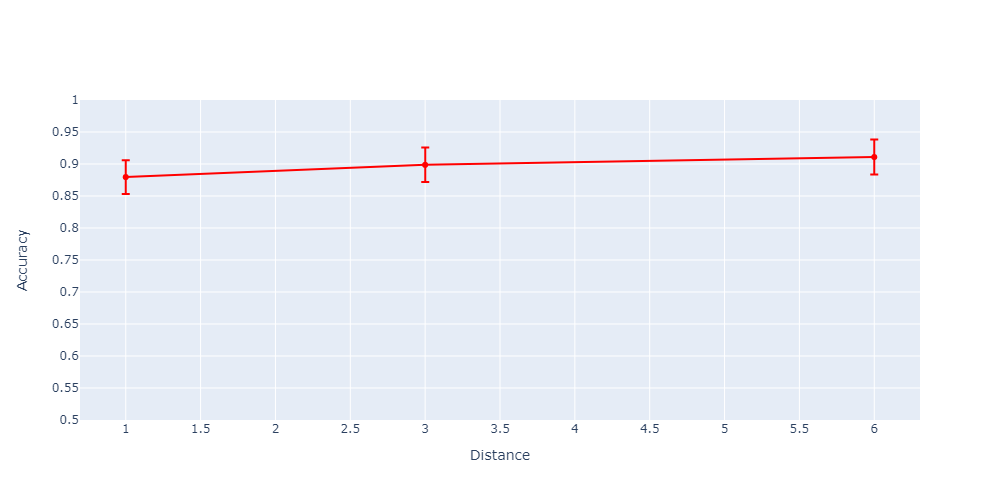


**Figure c-4.**  Weekend XGBoost - Analysis Window Size = 12 hours and Prediction Distance = 6 hours from drinking onset.

1. **General areas analyzed in the Two-Way Partial Dependence Plots (PDP) (Presented in Figure 9 in the manuscript)**


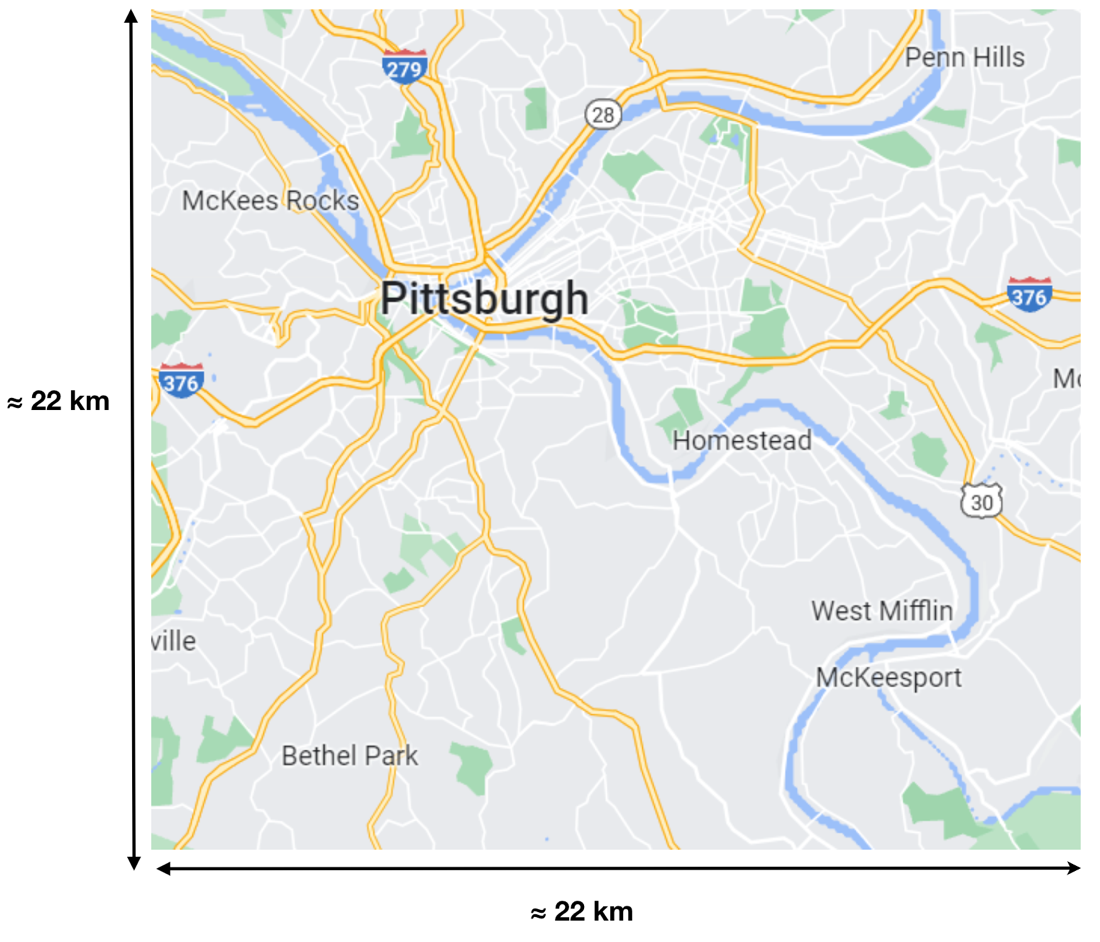


**Figure D-1. General Areas in the Pittsburgh Area that the samples were collected from, analyzed in the Two Way Partial Dependence Plots**

Contour plot analysis was done using the samples that fall inside the area represented in the map above for the weekdays model. Samples outside the Pittsburgh Metro Area were excluded from the analysis. Also excluded from the analysis were samples that could be classified as in Pittsburgh Metro Area, but that were spread sparsely and far away from the majority of the samples. The distribution of the samples mostly falls around the Three Rivers area.
